# Impact of glucocorticoid receptor polymorphism rs6198 on sepsis survival in a prospective multicenter cohort

**DOI:** 10.1038/s41598-025-07398-4

**Published:** 2025-07-09

**Authors:** Christoph Sombetzki, Lars Palmowski, Dominik Ziehe, Hartmuth Nowak, Tim Rahmel, Stefan Felix Ehrentraut, Patrick Thon, Jennifer Orlowski, Lars Bergmann, Michael Adamzik, Björn Koos, Katharina Rump, SepsisDataNet.NRW research group, Michael Adamzik, Moritz Anft, Thorsten Annecke, Nina Babel, Maha Bazzi, Lars Bergmann, Christian Bode, Thilo Bracht, Alexander von Busch, Jerome M. Defosse, Stefan F. Ehrentraut, Martin Eisennacher, Björn Ellger, Christian Ertmer, Ulrich H. Frey, Katrin Fuchs, Helge Haberl, Dietrich Henzler, Daniel Kleefisch, Thomas Köhler, Björn Koos, Ulrich Limper, Katrin Marcus, Hartmuth Nowak, Daniel Oswald, Christian Putensen, Tim Rahmel, Katharina Rump, Jens-Christian Schewe, Elke Schwier, Barbara Sitek, Matthias Unterberg, Frank Wappler, Katrin Willemsen, Alexander Wolf, Alexander Zarbock, Birgit Zuelch

**Affiliations:** 1 Department of Anesthesiology, Intensive Care and Pain Therapy, University Hospital Knappschaftskrankenhaus Bochum, Bochum, Germany; 2 Center for Artificial Intelligence, Medical Informatics and Data Science, University Hospital Knappschaftskrankenhaus Bochum, Bochum, Germany; 3 Department of Anesthesiology and Intensive Care Medicine, University Hospital Bonn, Bonn, Germany; 4 Klinik für Anästhesiologie, Intensivmedizin und Schmerztherapie, Universitätsklinikum Knappschaftskrankenhaus Bochum, Bochum, Germany; 5 Ruhr Universität Bochum, Medizinische Fakultät, Medizinisches Proteom-Center, Bochum, Germany; 6 Klinik für Anästhesiologie, Operative Intensivmedizin und Schmerztherapie, Universitätsklinikum Münster, Münster, Germany; 7 Klinik für Anästhesiologie und Operative Intensivmedizin, Universitätsklinikum Bonn, Bonn, Germany; 8 Klinik für Anaesthesiologie und Operative Intensivmedizin, Universität Witten/Herdecke, Krankenhaus Köln-Merheim, Köln, Germany; 9 Klinik für Anaesthesiologie und Operative Intensivmedizin, Rettungsmedizin und Schmerztherapie, Klinikum Herford, Herford, Germany; 10 Klinik für Anästhesiologie, Intensivmedizin und Schmerztherapie, Klinikum Westfalen, Dortmund, Germany; 11 Centrum für Translationale Medizin, Medizinische Klinik I, Marien Hospital Herne, Universitätsklinikum der Ruhr-Universität Bochum, Herne, Germany; 12 Klinik für Anästhesiologie, Operative Intensivmedizin, Schmerz- und Palliativmedizin, Marien Hospital Herne, Universitätsklinikum der Ruhr-Universität Bochum, Bochum, Germany

**Keywords:** Sepsis, Glucocorticoids, NR3C1, rs6198, Sepsis survival, Biomarker, Glucocorticoid receptor isoforms, Infectious diseases, Risk factors, Genetic markers, Genotype, Medical genetics

## Abstract

The glucocorticoid receptor (GR), particularly its isoforms GRα and GRβ, plays a crucial role in modulating inflammatory responses. The rs6198 single nucleotide polymorphism (SNP) in the NR3C1 gene, which encodes GR, has been associated with adverse outcomes in various diseases due to its potential effect on GR isoform expression. This study aims to explore the impact of the rs6198 SNP in sepsis. Specifically, we tested the hypothesis that the presence of a particular genotype of the rs6198 SNP is associated with an increased 30-day mortality rate in patients with sepsis. This prospective, multicenter study included 204 ICU patients diagnosed with sepsis, as part of the *Sepsis.Data.Net NRW* cohort. Genotyping for rs6198 and immunofluorescence as well as quantification of GR expression were performed. Statistical analyses included Hardy–Weinberg equilibrium, Kaplan–Meier survival analysis, log-rank tests, multivariate Cox regression, and logistic regression. Genotyping for the rs6198 SNP identified 137 patients (67%) with the TT- and 67 (33%) with CC/CT-genotype. Patients with the TT-genotype had a 30-day survival rate of 65% (89 of 137 patients), which was significantly lower than the 82% survival rate (55 of 67 patients) observed in the patients with the CC/CT-genotype (*p* = 0.006). A multivariate Cox regression analysis, adjusted for age, SOFA and SAPS2 score, and selected laboratory values, revealed that the TT-genotype was independently associated with an increased risk of death (HR 3.56, 95% CI 1.22–10.38, *p* = 0.02). Subgroup analysis demonstrated a particularly pronounced impact among patients with initially high disease severity (HR 6.16, 95% CI 1.66–22.80, *p* = 0.007). In addition, expression analysis revealed a significantly higher presence of GRα in patients with the TT-genotype compared to those with CC/CT genotype (*p* = 0.023). Increased GRα expression was also associated with higher 30-day mortality (HR 2.38, 95% CI 1.48–3.82, *p* < 0.001). The rs6198 SNP in the NR3C1 gene is associated with 30-day mortality in sepsis patients and correlates with increased expression of the GRα isoform. These results highlight the TT-genotype as a potential risk marker. Further research is needed to clarify the causal mechanisms and explore personalized therapeutic implications in sepsis management.

## Introduction

Sepsis is a life-threatening condition characterized by organ dysfunction due to a dysregulated immune response to an infection^1^. Current research efforts are directed towards identifying key proteins that decisively influence clinical outcomes, potentially guiding therapeutic interventions towards recovery or mitigating mortality^2–4^. One important system that modulates inflammation in sepsis is the glucocorticoid system. Within this system, polymorphisms in key proteins appear to influence the response to glucocorticoid therapy. In this context, we have previously investigated single nucleotide polymorphism (SNPs) in glucocorticoid-induced leucine zipper (GILZ)^6,7^ and NF-κB^5^, both of which were associated with significant effects on treatment response. However, the central protein of this system, the glucocorticoid receptor (GR), has received relatively little attention^5^. Given its pivotal role in the immune response to sepsis^8^, a deeper understanding of genetic variants affecting GR function is warranted while it regulates a wide array of pro- and anti-inflammatory signaling pathways^6^. The receptor exists in two primary isoforms: the alpha isoform (GRα) and the beta isoform (GRβ)^7^. GRα generally exerts anti-inflammatory effects by inducing transcription of genes such as GILZ and monocyte chemotactic protein (MCP-1), and by inhibiting the activation of pro-inflammatory mediators such as the p65 subunit of NFκB^8,9^. In contrast, GRβ tends to enhance inflammatory responses due to its antagonistic interaction with GRα^10^. Considerable attention has been paid to genetic variations within the NR3C1 gene, in particular the SNP rs6198 ^11–14^. This SNP has been associated with adverse outcomes in immune-mediated diseases such as rheumatoid arthritis and post-traumatic stress disorder, mainly through its influence on mRNA stability, which favors the pro-inflammatory GRβ isoform^15,16^. However, the impact of rs6198 SNP within NR3C1 in sepsis, specifically its effects on the differential expression of GRα versus GRβ, and consequently on sepsis outcomes, has not been investigated. Therefore, we tested the hypothesis that the presence of a particular genotype of the rs6198 SNP is associated with an increased 30-day mortality rate in patients with sepsis.

## Materials and methods

### Study design and conceptual overview

204 patients from the prospective, multicenter Sepsis.Data.Net NRW cohort were included. Patients were recruited consecutively between 2018 and 2020. Systematic screening ensured that all eligible ICU patients were considered for inclusion (see supplementary file 1). Written consent was obtained from all patients or their legal guardians. This study was approved by the Ethics Committee of the Medical Faculty of the Ruhr-University of Bochum (Registration no. 19–6606 3-BR). All research involving human participants was conducted in full accordance with the Declaration of Helsinki, as well as institutional and national guidelines and regulations. The study strictly adhered to the protocols outlined in the approved ethics vote. Intensive care patients aged 18 and older were eligible for recruitment if they met the current Sepsis-3 criteria for sepsis diagnosis^1^. To enhance the generalizability of our findings and account for the heterogeneity of sepsis progression, the study protocol allowed for patient inclusion within 48 h of sepsis diagnosis. This ensured that patients initially treated on the general ward before ICU transfer were not systematically excluded. For patients diagnosed with sepsis upon ICU admission, enrollment and sample collection were performed immediately to capture the earliest possible disease stage. Treatment of patients was carried out according to the current national and international guidelines and was not influenced by participation in the study^17^. Hydrocortisone therapy was defined as 200 mg/24 h via infusion pump for at least 24 h, according to guidelines^17^. Blood samples for DNA, RNA, and serum analysis were collected within the first 48 h after diagnosis and stored at − 80 °C after initial processing.

### DNA genotyping

DNA was isolated from EDTA-blood samples using the my-Budget Blood DNA Midi Kit (Bio-Budget Technologies GmbH, Krefeld, Germany) according to the manufacturer’s instructions as previously described^18^. Genotyping was performed using the Thermo Fisher Scientific TaqMan® SNP Genotyping Assay (Thermo Fisher Scientific, Wilmington, USA) and Bio-Rad CFX Connect Cycler Systems (Bio-Rad Laboratories, Inc., Hercules, USA) using a protocol of 95 °C for 10 min and 40 cycles of 95 °C for 15 s followed by 60 °C for 60 s.

### RNA analysis

RNA was extracted from whole blood collected with Tempus™ Blood RNA Tubes (Applied Biosystems, Waltham, USA) using Tempus™ Spin RNA Isolation Reagent Kits (Applied Biosystems, Waltham, USA), followed by complementary DNA (cDNA) synthesis using the High-Capacity cDNA Reverse Transcription Kit by Applied Biosystems (Applied Biosystems, Waltham, USA). Then, quantitative polymerase chain reaction was performed using our primers listed in supplementary file 2 for expression analysis of total GR, GRα and GRβ in relation to ACTB, as proposed^19^. The protocol used with GoTaq® qPCR MasterMix (Promega, Madison, USA) involved 2 min of 95 °C followed by 40 cycles of 95 °C for 15 s and 60 °C for 60 s.

### Cortisol and GILZ, MCP-1 concentrations

Serum samples were analyzed using enzyme-linked immunosorbent assay (ELISA) kits to determine cortisol, MCP-1, and GILZ levels (Enzo Life Sciences, Inc., Farmingdale, USA; Antibodies-online GmbH, Aachen, Germany). Samples were diluted as necessary to fall within the standard detection range of each kit. Blood samples were consistently drawn at 6 AM to account for potential circadian variations in cortisol concentrations.

### Cytokine measurements

The concentrations of various cytokines were quantified on day one using a customized human LegendPlex assay (BioLegend, San Diego, CA). The cytokines measured included Interleukin-1 beta (IL-1β), Interleukin-6 (IL-6), Interleukin-10 (IL-10), Interleukin-12 (IL-12), Interleukin-18 (IL-18), Interleukin-23 (IL-23), tumor necrosis factor alpha (TNF-α), Interferon alpha2 (INF-α2), and Interferon gamma (INF-γ),

### Cytospin and immunofluorescence

Peripheral blood mononuclear cells (PBMCs) from septic patients were collected on day one of sepsis and centrifuged onto glass slides using a Cellspin device (Tharmac, Limburg, Germany), fixed with 4% formaldehyde (F1635, Sigma Aldrich, Taufkirchen, Germany) and permeabilised with 0.1% (v/v) Triton-X100 (T8787, Sigma Aldrich) and 0.1% (m/v) sodium dodecyl sulfate (SDS) (0183.1, Carl Roth, Karlsruhe, Germany). Non-specific binding sites were blocked using Duolink® blocking solution (DUP82007, Sigma Aldrich). The slides were then incubated overnight at 4 °C with a mouse anti-human GR antibody (Glucocorticoid Receptor (D4X9S) Mouse mAb #47,411). After washing, the slides were incubated for 1 h at 37 °C with goat anti-mouse IgG AlexaFluor® 488 (1:400 in PBS, ab150077, Abcam, Cambridge, UK). Counterstaining was performed using SlowFade™ Gold Antifade Mountant with DAPI (S36939, Invitrogen) and phalloidin solution. Fluorescence microscopy was carried out using an Olympus IX51 microscope (Olympus, Hamburg, Germany). All samples were imaged and analyzed under consistent settings using Fiji ImageJ software.

### Statistical analysis

Continuous variables are presented as means ± standard deviation (SD) when normally distributed and as medians with interquartile ranges (IQR; 25th to 75th percentile) for distributions that are not normal. Differences between groups for continuous data were determined using the t-test or the Wilcoxon rank-sum test, depending on the distribution of the data. For categorical variables, differences between groups were evaluated using either the Chi-square test or Fisher’s exact test, as appropriate. The distribution of genotypes were tested for Hardy–Weinberg equilibrium to ensure that genetic variation was consistent with expected frequencies using a chi-squared test. Survival curves were constructed using the Kaplan–Meier method to estimate the time-dependent probability of survival. The log-rank test was applied to statistically compare these curves between the different groups and to identify significant differences in survival time. For the multivariable regression analysis, we applied a structured feature selection approach to ensure robustness and prevent overfitting. Univariate analysis was first conducted on the rs6198 SNP, key clinical variables (SOFA/SAPS II scores), and laboratory markers. Statistically significant variables were considered for the final model, with collinearity addressed by selecting the strongest predictor among correlated variables. To adhere to standard guidelines, we included a maximum of six variables for our 204-patient cohort. The full univariate analysis is provided in the supplementary material (see supplementary file 3). Multivariate Cox regression analysis was used to evaluate the influence of various predictors on survival, adjusting for potential confounders and quantifying hazard ratios. We assessed interaction effects within a generalized linear model by including interaction terms between the rs6198 TT genotype and both SOFA Score ≥ 9 and hydrocortisone therapy to evaluate whether the association with 30-day mortality varied across these subgroups. To assess outcomes in patients with more severe illness, we defined a high-severity subgroup based on a SOFA score ≥ 9. This threshold was chosen as it exceeds the median SOFA score of 8 in our cohort, allowing us to focus on patients with more pronounced organ dysfunction. Unlike septic shock alone, which is already partly reflected in the cardiovascular SOFA subcomponent, this approach provides a broader measure of disease severity, incorporating dysfunction across multiple organ systems. A *p*-value of less than 0.05 was deemed to indicate statistical significance. Confidence intervals (CIs) were set at 95% coverage. All statistical analyses were conducted using R software (version 3.5.3; The R Foundation for Statistical Computing; http://www.R-project.org).

## Results

### Baseline characteristics

In all 204 patients, genotyping of the rs6198 NR3C1 was performed. A total of 137 patients exhibited the TT-genotype. The genotype distribution was in Hardy–Weinberg equilibrium (*p* = 0.892) and the less frequent CC-genotype (n = 7) was pooled with the heterozygous CT-genotype (n = 60) for further analyses. There was no significant difference between the TT and CC/CT genotype groups in terms of gender, age and SOFA score at enrollment, as shown in Table 1. Similarly, no significant differences were found in terms of infection focus, length of stay in the ICU. Regarding laboratory variables at admission, the CC/CT-genotype group exhibited higher bilirubin concentrations (0.77 mg/dL, IQR: 0.41–1.64) compared to the TT-genotype group (0.52 mg/dL, IQR: 0.32–0.91, *p* = 0.043).

**Table 1:** Cohort description, classification according to rs6198 SNP TT and CC/CT-genotype.

	TT (n = 137)	CC/CT (n = 67)	*p*-value
Base characteristics			
Female sex, n (%)	55 (40,88%)	20 (30,77%)	0.167
Age, years (IQR)	64.0 (56–74)	63 (54–74)	0.426
BMI, kg/m^2^ (IQR)	24.8 (23.1–30.8)	27.6 (24.2–30.4)	0.635
SAPSII Score, day 1 (IQR)	31.5 (26.0–38.0)	33.0 (22.0–41.0)	0.765
SOFA Score, day 1 (IQR)	8.0 (5.0–11.0)	8.0 (5.0–10.5)	0.630
Septic Shock, day 1, n (%)	46 (34%)	26 (39%)	0.594
Ventilatory support*, day 1, n (%)	112 (86%)	52 (87%)	1.000
Length of ICU stay, days (IQR)	6.61 (2.52–12.08)	7.34 (3.77–13.78)	0.250
Comorbid conditions, n (%)			
Hypertension	83 (61%)	31 (46%)	0.142
Cardiovascular disease	42 (31%)	20 (30%)	0.863
Obesity**	30 (22%)	10 (15%)	0.676
COPD***	19 (14%)	7 (10%)	0.818
Diabetes mellitus	40 (29%)	11 (16%)	0.101
Chronic kidney disease	24 (18%)	13 (19%)	0.551
Malignant neoplasms	30 (22%)	9 (13%)	0.241
Infection focus, n (%)			
Pulmonal	59 (43.1%)	29 (43.3%)	0.636
Urinary tract	8 (5.8%)	5 (7.5%)	
Abdomen	20 (14.6%)	6 (9.0%)	
Central nervous system	1 (0.7%)	2 (3,0%)	
Bloodstream	9 (6.6%)	2 (3.0%)	
Skin & Soft Tissue	12 (8.8%)	7 (10.4%)	
Other/Unknown	28 (20.4%)	16 (23.9%)	
Laboratory values, day 1			
C-reactive protein [mg/L]	15.43 (8.92–25.72)	18.13 (10.34–28.73)	0.423
Procalcitonin [ng/mL]	2.12 (0.35–12.76)	4.91 (0.40–11.37)	0.900
Leucocytes [1000/µL]	13.25 (9.33–18.80)	11.75 (8.95–16.33)	0.236
Hemoglobin [g/dL]	8.95 (7.7–9.5)	8.65 (7.8–11.0)	0.653
Lactate [mmol/L]	1.30 (0.90–2.00)	1.20 (0.85–1.55)	0.187
INR [—]	1.18 (1.12–1.26)	1.29 (1.17–1.42)	0.016
Fibrinogen [mg/dl]	516.0 (350.2–587.8)	520.5 (454.0–656.5)	0.495
ALAT [U/L]	17.6 (9.1–76.4)	24.3 (12.95–46.41)	0.813
ASAT [U/L]	32.5 (15.6–121.6)	43.7 (18.2–87.4)	0.734
Potassium [mmol/L]	4.7 (4.2–5.1)	4.5 (4.3–5.2)	0.780
Creatinine [mg/dL]	1.14 (0.88–1.79)	1.22 (0.84–2.01)	0.924
Bilirubin [mg/dL]	0.52 (0.32–0.91)	0.77 (0.41–1.64)	0.043

Data are presented as n (%) and median (IQR). *Ventilatory support included: CPAP / High flow / Invasive ventilation **defined as BMI ≥ 30 kg/m^2^ ***Chronic obstructive pulmonary disease (COPD).

### The rs6198 SNP association with 30-day survival

**Fig. 1:**
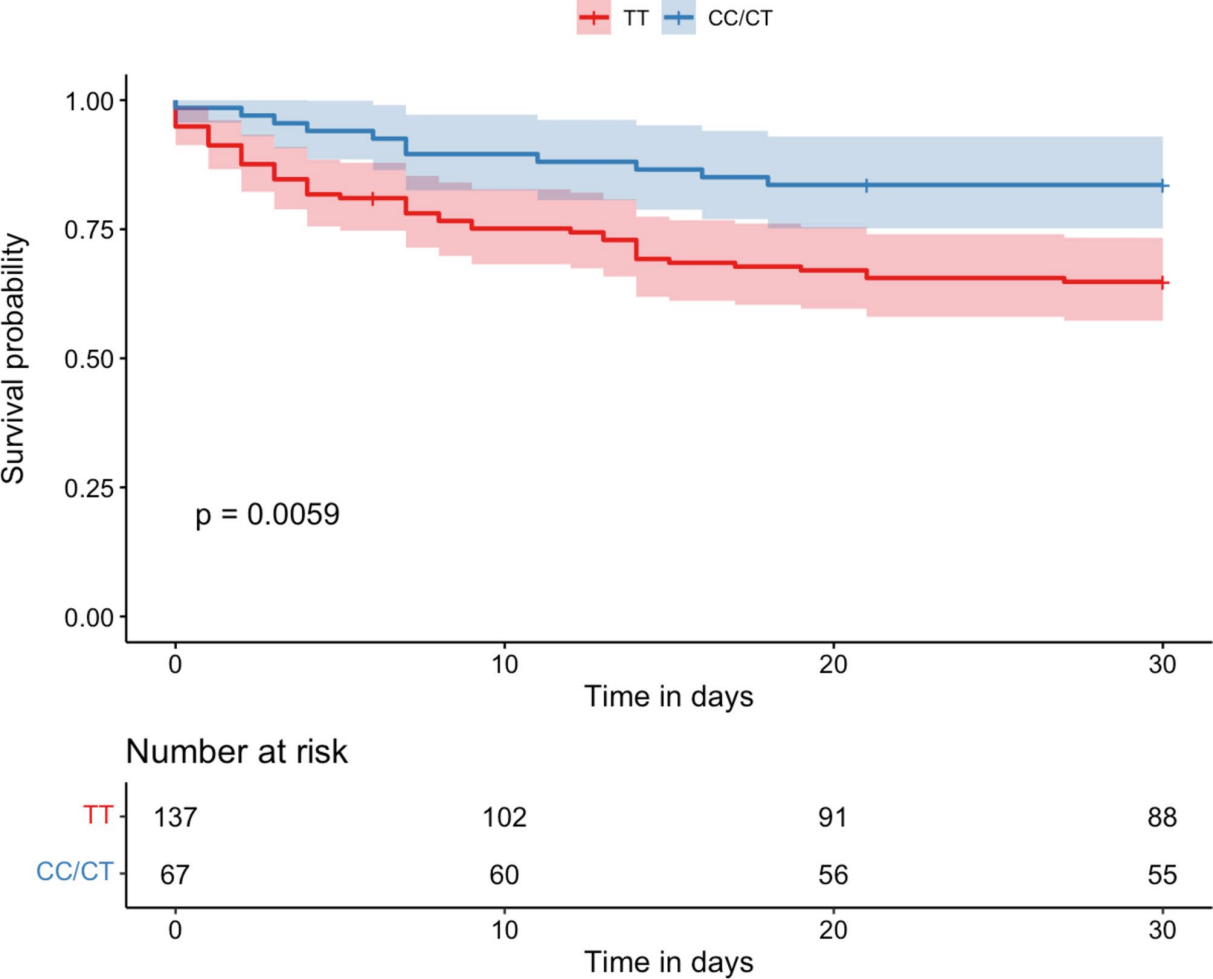
Kaplan–Meier Survival Analysis of the entire Cohort (n = 204 patients) based on rs6198 SNP genotypes. This figure displays the survival curves for patients stratified by TT and CC/CT genotypes of the rs6198 SNP. Patients with the TT-genotype exhibit significantly higher 30-day mortality compared to those with the CC/CT-genotype.

Carriers of the TT-genotype demonstrated a 30-day survival rate of 65% (89 out of 137 patients). In contrast, individuals with CC/CT genotype had a significantly higher 30-day survival rate of 82% (55 out of 67 patients, *p* = 0.006), as illustrated in Fig. 1.

In the multivariate Cox regression analysis, we adjusted the rs6198 genotype for additional potential confounding variables such as age and the SOFA score on day one. Further adjustments were made for the administration of hydrocortisone during the ICU stay, serum bilirubin concentrations, which differed significantly between the groups (see above) and serum lactate concentrations. The TT-genotype remained a significant, independent risk factor, with a hazard ratio of 3.56 (95% CI 1.22–10.38, *p* = 0.02), as demonstrated in Table 2.

**Table 2:** Cox regression analysis for 30d mortality of septic patients (n = 204).

	Variable	Univariate		Multivariate	
		HR	*p*-value	HR	*p*-value
Base characteristics	rs6198 Genotype TT*	2.44 (1.27–4.69)	**0.008**	3.56 (1.22–10.38)	**0.020**
	Age	1.02 (1.00–1.04)	**0.031**	1.01 (0.97–1.05)	0.654
	SOFA Score, day 1	1.34 (1.25–1.45)	** < 0.001**	1.13 (0.99–1.29)	0.072
	SAPS2, day 1	1.09 (1.05–1.14)	** < 0.001**	1.06 (1.99–1.12)	0.066
	Hydrocortisone therapy**	2.05 (1.22–3.47)	**0.007**	2.16 (0.88–5.28)	0.092
Lab values	Serum lactate (mg/dL)	1.31 (1.22–1.41)	** < 0.001**	1.15 (1.01–1.31)	**0.035**
	Bilirubin (mg/dL)	1.31 (0.96–1.80)	0.090	1.61 (0.96–2.68)	0.069

Multivariate cox regression with Hazard ratios and 95% CI Intervals, *Genotype of rs6198-SNP in NR3C1-Gene ** during ICU stay, as described in methods.

Significant values are in bold.

The interaction analysis revealed a significant modification of the association between the rs6198 TT genotype and 30-day mortality by SOFA Score ≥ 9 (HR: 1.43, *p* = 0.003) and hydrocortisone therapy (HR: 1.19, *p* = 0.021), as detailed in supplementary file 4. These effects were further explored within the following subgroup analysis.

### The influence of SNP rs6198 depending on disease severity

**Fig. 2:**
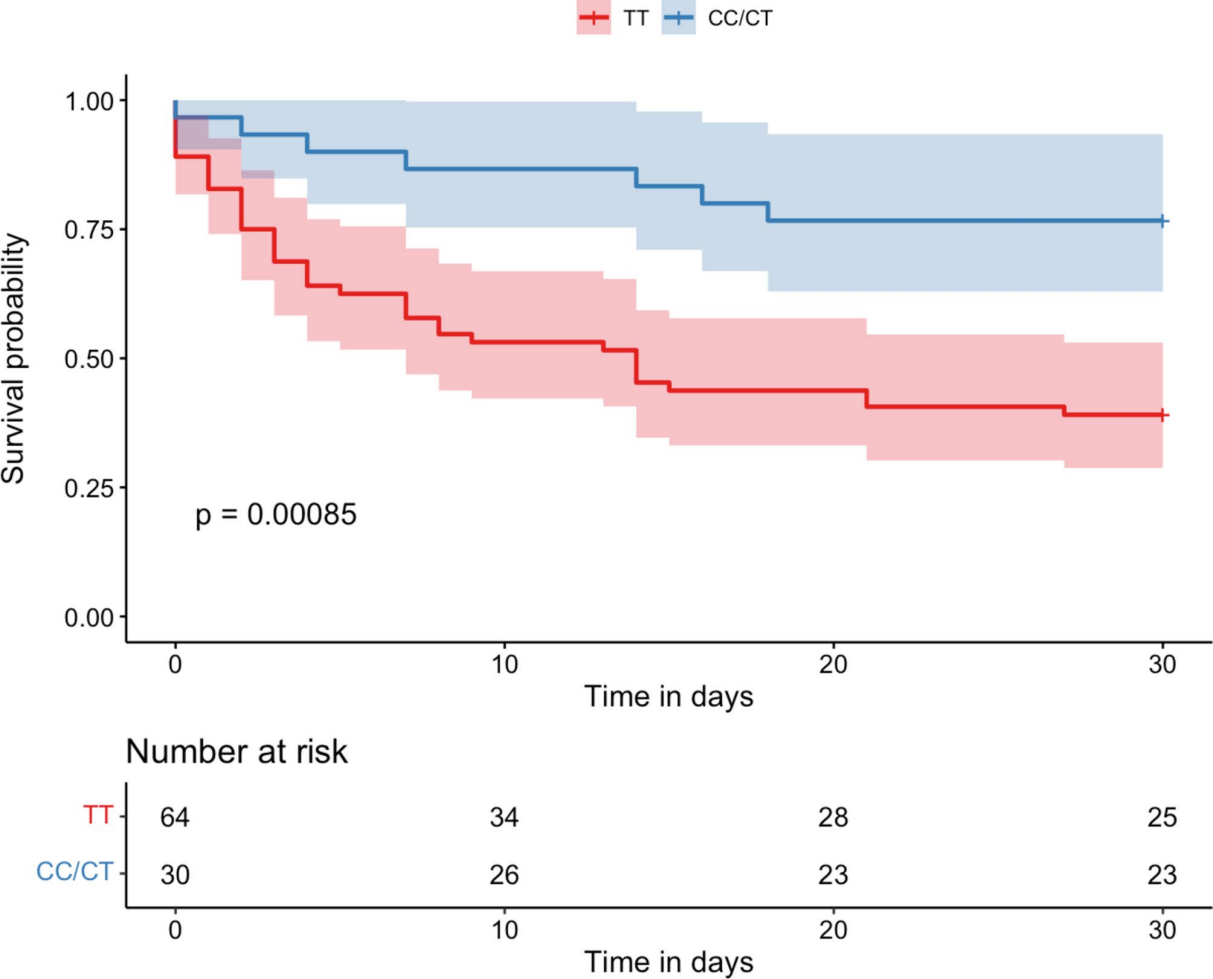
Kaplan–Meier Survival Analysis for the Subgroup with Septic Shock or SOFA Score ≥ 9 at Enrollment (n = 94) Based on rs6198 SNP Genotypes. This figure presents survival curves for patients with higher disease severity, stratified by TT and CC/CT genotypes of the rs6198 SNP. The increased 30-day mortality associated with the TT genotype observed in the entire cohort (see Fig. 1) is more pronounced in this subgroup analysis.

To analyze the extent to which the described effects impact 30-day survival in patients with higher disease severity (i.e., presence of septic shock within 24 h of study enrollment or a SOFA score of ≥ 9 at study entry), we conducted a subgroup analysis. Of the 204 patients, 94 fell into this subgroup (46%) and their baseline characteristics are available in supplementary file 5. The association of TT-genotype with a higher mortality rate was even more pronounced in this subgroup with greater disease severity, as shown in Fig. 2. Performing the previously described multivariate Cox regression in this subgroup, the presence of a TT-genotype resulted in a hazard ratio of 6.16 (95% CI 1.66–22.80, *p* = 0.007; see supplementary File 6 for the complete analysis). No significant effects were observed in the corresponding subgroup (i.e. SOFA score < 9 and no septic shock) (see supplementary File 7).

### The influence of SNP rs6198 in relation to hydrocortisone therapy

Within the entire cohort, 53 patients (26%; TT-genotype 40 out of 53 patients vs. CC/CT-genotype 13 out of 53 patients) received hydrocortisone therapy during their sepsis management in the intensive care unit. The mortality rate among all patients undergoing hydrocortisone therapy was 43% (23 out of 53 patients), and thus significantly higher than in patients who did not receive hydrocortisone therapy (25%, 38 out of 151 patients, *p* = 0.006). In patients treated with hydrocortisone, no statistically significant association was observed between SNP rs6198 and 30-day mortality (TT-genotype 48%, 19 out of 40 patients vs. CC/CT-genotype 31%, 4 out of 13 patients, *p* = 0.34). In contrast, among patients not receiving hydrocortisone therapy, the TT-genotype was associated with a significantly higher mortality rate (TT-genotype 31%, 30 out of 97 patients vs. CC/CT-genotype 15%, 8 out of 54 patients, *p* = 0.017).

### The rs6198 SNP association with GR expression

**Fig. 3:**
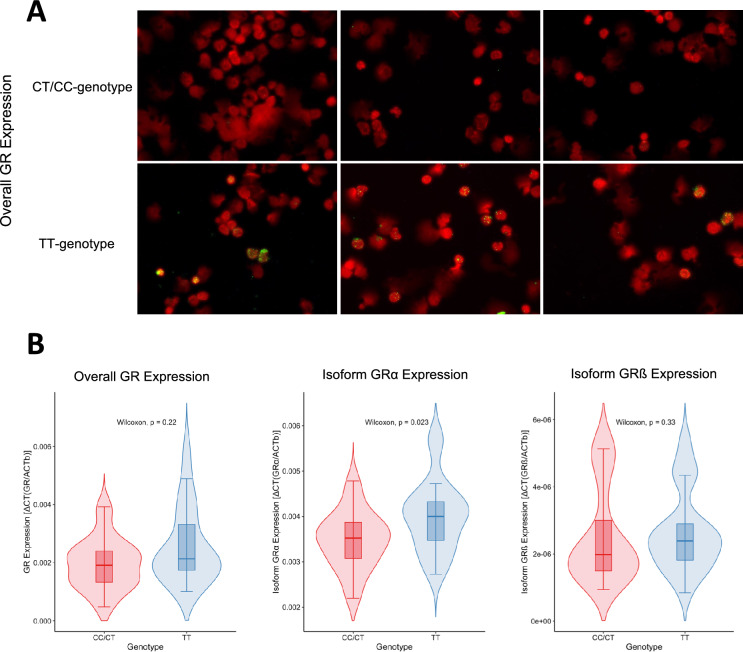
Qualitative and Quantitative Expression Analysis of GR and Its Isoforms Alpha and Beta in PBMCs of Septic Patients. **A** Immunofluorescence depiction of overall GR protein (green dots) alongside actin filaments, stained with phalloidin (red). **B** Quantitative mRNA expression of GR and its isoforms, alpha and beta. While no significant differences in overall GR expression are observed between patients possessing TT and CC/CT genotypes of the rs6198 SNP, there is a significant increase in GRα expression associated with the TT genotype.

We conducted a qualitative assessment of GR protein expression In the immunofluorescence staining of PBMCs from septic patients. Notably, individuals with TT genotypes showed an enhanced GR expression signal, as illustrated in Fig. 3A. However, subsequent RNA analysis revealed no significant differences in overall GR expression between the TT and CC/CT genotypes (*p* = 0.220), as shown in Fig. 3B. Further analyses were performed separately for the alpha and beta isoforms of GR. The TT-genotype was significantly associated with increased expression of GRα (*p* = 0.023), while GRβ expression showed no significant differences (*p* = 0.330). We also investigated the impact of GR expression on 30-day mortality using COX- regression. Increased GRα expression was significantly associated with higher 30-day mortality (HR 2.38, 95% CI 1.48–3.82, *p* < 0.001). In contrast, for GRβ (HR 1.00, 95% CI 1.00–1.00, *p* = 0.211) and overall GR expression (HR 1.21, 95% CI 0.81–1.83, *p* = 0.352), no association with 30-day mortality was observed.

### Analysis of downstream proteins GILZ and MCP-1, serum cortisol levels and cytokines

As indicated in Table 3, there were no significant differences in the expression levels of the downstream proteins MCP-1 (*p* = 0.449) and GILZ (*p* = 0.115) between the TT-genotype and the CC/CT genotype, when considering the entire cohort. Additionally, the overall serum cortisol concentrations did not demostrate significant differences between these subgroups (*p* = 0.733). Similarly, cytokine measurements showed no significant differences between the TT genotype and the CC/CT genotypes (all *p*-values exceeding 0.05). However, again when examining the subgroup defined by higher disease severity—in particular, those with septic shock or a SOFA score ≥ 9 —the TT genotype was associated with significantly higher concentrations of the cytokines IL-12, IL-23, and IFN-γ (each *p*-values below 0.05).

**Table 3:** Expression analysis of downstream proteins, cortisol and cytokines.

Variable	TT (n = 137)	CC/CT (n = 67)	*p*-value
Overall cohort			
Expression analysis			
GILZ*, pg/ml (IQR)	0.054 (0.022–0.092)	0.029 (0.019–0.047)	0.114
MCP-1**, pg/ml (IQR)	133.80 (88.01–208.57)	118.73 (62.54–184.38)	0.449
Cortisol analysis			
Serum cortisol, ng/ml (IQR)	5.50 (3.61–6.86)	5.48 (4.17–6.52)	0.733
Cytokine measurements			
IL-6, pg/ml (IQR)	176.99 (52.54–491.33)	215.65 (83.53–539.77)	0.570
IL-10, pg/ml (IQR)	7.06 (2.31–17.23)	6.57 (2.22–13.16)	0.477
IL-12, pg/ml (IQR)	2.98 (2.06–4.47)	2.82 (1.83–4.16)	0.169
IL-23, pg/ml (IQR)	13.12 (0.00–28.19)	6.52 (0.00–27.48)	0.240
TNF-α, pg/ml (IQR)	6.65 (2.40–10.22)	5.43 (0.00–9.13)	0.603
INF- γ, pg/ml (IQR)	3.79 (0.00–7.62)	4.56 (1.69–8.94)	0.327

Variable	TT (n = 64)	CC/CT (n = 30)	*p*-value
Subgroup with septic shock or SOFA ≥ 9***			
IL-12, pg/ml (IQR)	2.94 (2.40–4.33)	2.43 (1.68–3.15)	0.036
IL-23, pg/ml (IQR)	16.64 (6.52–41.13)	6.33 (0.00–18.41)	0.009
INF- γ, pg/ml (IQR)	5.18 (2.44–8.54)	2.27 (0.00–5.10)	0.022

Data analysed on day 1, presented as median (IQR). *Glucocorticoid-Induced Leucine Zipper (GILZ). **Monocyte Chemoattractant Protein-1 (MCP-1), ***Only cytokines with significant group differences are listed in the results. All other variables not mentioned exhibited a *p*-value greater than 0.05.

## Discussion

Our study demonstrates that the presence of the NR3C1 rs6198 SNP influences survival in sepsis. In particular, the presence of the TT-genotype is associated with significantly higher mortality, especially in patients with initially severe disease states.

Upon genotyping our cohort, we observed a TT to CC/CT ratio of 2:1, which is consistent with previous literature^12,15^. At baseline, there were no other significant baseline characteristic differences or disease severity factors at study entry that could have influenced the outcomes. Nevertheless, both the Kaplan–Meier analysis and the Cox regression, adjusted for potential confounders, confirmed the TT-genotype as an independent risk factor for increased mortality. This raises questions about the underlying mechanisms involved.

First, we investigated whether the TT genotype influences GR expression. Initially, immunofluorescence suggested an increased overall expression of GR. Unfortunately, our subsequent quantitative analyses did not achieve statistical significance, possibly due to the small sample size. However, the situation changed when we examined the GR isoforms separately. In this analysis, carriers of the TT genotype exhibited significantly higher expression of the alpha isoform. As previously noted, the alpha isoform is crucial for the anti-inflammatory effects of cortisol^5,20^. In our cohort, increased expression of GRα was significantly associated with higher mortality, suggesting that the rs6198 SNP may influence sepsis survival through this mechanism. However, the literature shows heterogeneous results regarding GR isoforms, particularly in studies involving critically ill children^5,21–23^. Further confirmatory studies are needed to clarify the impact of increased GRα expression.

Second, we observed enhanced mortality effects of the TT-genotype in a subgroup with particularly high disease severity, such as septic shock. This group is inherently vulnerable and characterized by high mortality rates^24^. Given that guideline-adherent hydrocortisone therapy may be considered in this subgroup, we intentionally focused our analysis on these patients.^17^. We specifically examined whether hydrocortisone therapy differentially impacts carriers of the TT versus CC/CT-genotype. In patients receiving hydrocortisone therapy, no significant differences regarding 30-day mortality were observed. Conversely, in the subgroup without hydrocortisone, TT-genotype carriers died significantly more frequently than CC/CT carriers. Given our study’s observational design, it remains an open question whether TT-genotype would have benefited from hydrocortisone therapy in sepsis^25^. Further research is needed to determine if this could be an avenue for personalized therapy based on patients’ genotype^26^.

Third, our work examines the rs6198 SNP’s influence on downstream proteins such as GILZ, MCP-1 and various cytokines and examines whether serum cortisol itself could be responsible for the differences. Here, we found no significant group differences concerning TT or CC/CT-genotype, when considering the entire cohort suggesting that the observed effects might be explained by other mechanisms. However, a distinct pattern emerged when analyzing the above described subgroup characterized by high disease severity. In this subgroup, in addition to a more pronounced association between the TT genotype and increased 30-day mortality, significant increases in IL-12, IL-23, and IFN-γ were also observed. IL-12 plays a crucial role in the differentiation of naive T cells into Th1 cells^27^. It serves as a T cell activator, facilitating the proliferation and activation of T cells. More specifically, IL-12 induces the secretion of INF-γ and TNF-α from T cells and natural killer cells, while concurrently countering the inhibitory impact of IL-4 on IFN-γ production^28^. Similarly, IL-23 stimulates the production of IFN-γ and enhances the proliferation of activated T cells^29^. These actions highlight the essential roles of IL-12 and IL-23 in bolstering T-cell mediated immune responses.

Considering these cytokine profiles alongside our genetic findings, it appears that the rs6198 SNP, by influencing higher levels of GRα, especially in critically ill patients, correlates with enhanced cytokine release and augmented T-cell function. This association suggests that GRα may exert a modulatory role on T-cell responses, potentially impacting the efficacy of immune responses. Such findings emphasize the intricate interplay between genetic variations and immune system regulation, offering potential insights into tailored therapeutic approaches based on genetic predispositions.

## Limitations

Our study has several limitations. Although our cohort was multicentric and recruited prospectively, the observational study design does not establish causality between the presence of the TT-genotype and increased mortality in sepsis. Moreover, it is beyond the scope of our study to elucidate all potential effects of the rs6198 SNP. Future research investigating GR receptor activity, further downstream proteins in their varying expressions, and longitudinal measurements during sepsis may provide a more comprehensive understanding of the rs6198 SNP’s impact on sepsis survival. Additionally, while the overall cohort size was sufficient for the primary analysis, the sample size within subgroups was smaller, which may have limited statistical power to detect more subtle but potentially clinically relevant associations. This increases the risk of type II errors and necessitates cautious interpretation of the interaction effects observed.

## Conclusion

The TT genotype of rs6198 SNP in the NR3C1 gene is associated with higher 30-day mortality in sepsis patients and increased expression of the GRα isoform. Further studies are required to clarify the causal mechanisms and to determine if this represents a potential marker for a personalized therapy in sepsis.

## Supplementary Information

Supplementary Information 1.

Supplementary Information 2.

Supplementary Information 3.

Supplementary Information 4.

Supplementary Information 5.

Supplementary Information 6.

Supplementary Information 7.

## Data Availability

The datasets used and analyzed during the study are available from the corresponding author on reasonable request.

## Abbreviations

cDNA: Complementary DNA
CI: Confidence interval
DNA: Deoxyribonucleic acid
ELISA: Enzyme-linked immunosorbent assay
GILZ: Glucocorticoid-induced leucine zipper
GR: Glucocorticoid receptor
HR: Hazard ratio
IQR: Interquartile range
MCP-1: Monocyte chemoattractant protein-1
NFκB: Nuclear factor kappa-light-chain-enhancer of activated B cells
NR3C1: Nuclear receptor subfamily 3 group C member 1
qPCR: Quantitative polymerase chain reaction
RNA: Ribonucleic acid
SD: Standard deviation
SNP: Single nucleotide polymorphism
