# Supplementary figures and images for: Impact of glucocorticoid receptor polymorphism rs6198 on sepsis survival in a prospective multicenter cohort
Source: Sci Rep. 2025 Jul 9;15:24760. doi: 10.1038/s41598-025-07398-4 (PMC12241491; doi:10.1038/s41598-025-07398-4)

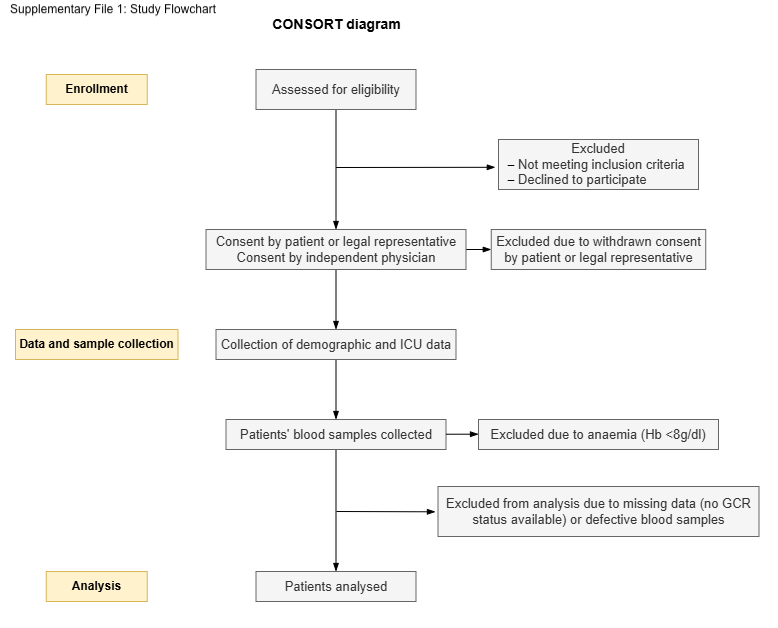

Supplement: Supplementary file 1 — Supplementary Information 1. [file 41598_2025_7398_MOESM1_ESM.png]

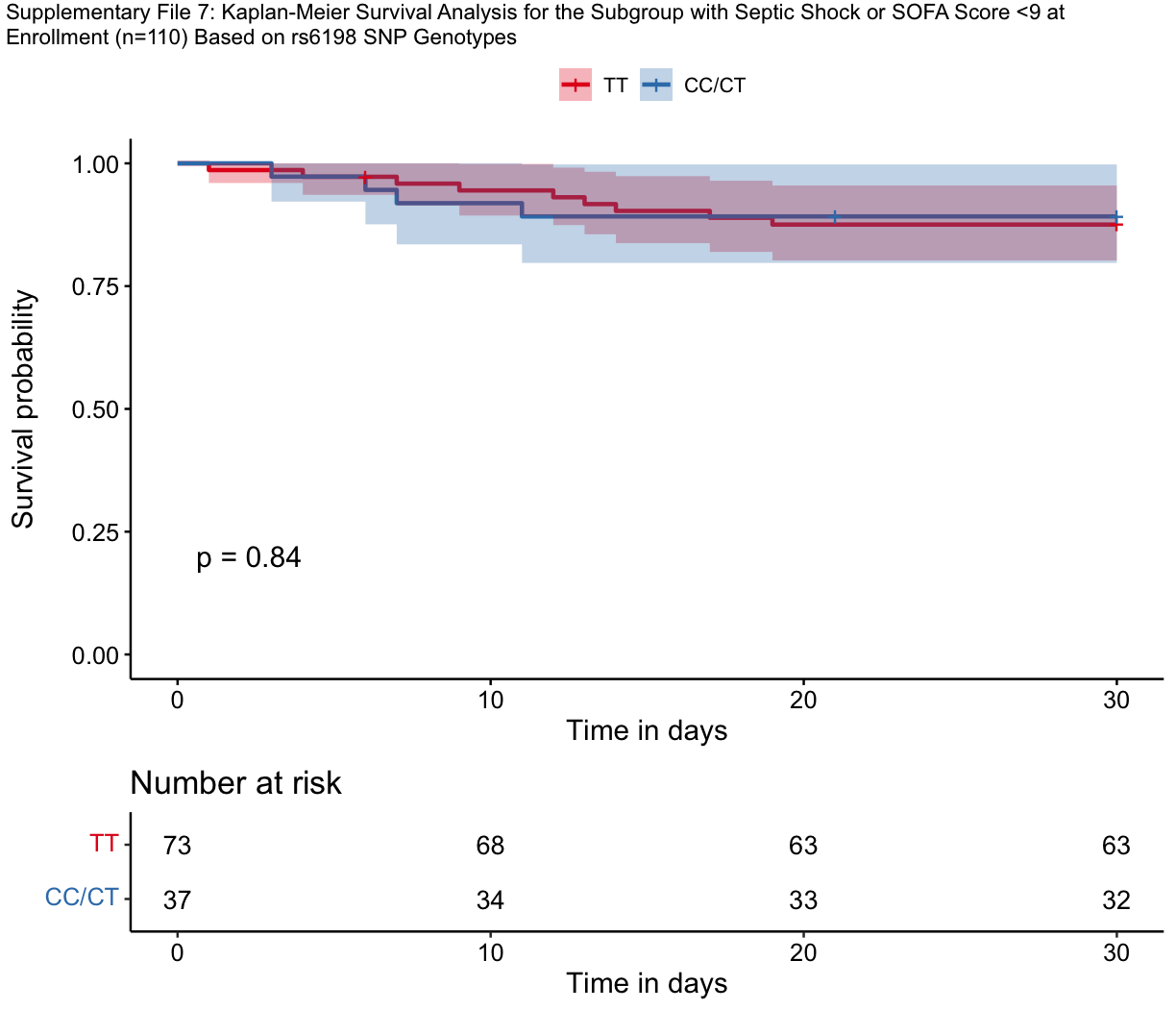

Supplement: Supplementary file 7 — Supplementary Information 7. [file 41598_2025_7398_MOESM7_ESM.png]
