# Supplementary material for: Impact of glucocorticoid receptor polymorphism rs6198 on sepsis survival in a prospective multicenter cohort
Source: Sci Rep. 2025 Jul 9;15:24760. doi: 10.1038/s41598-025-07398-4 (PMC12241491; doi:10.1038/s41598-025-07398-4)
Supplement: Supplementary file 2 — Supplementary Information 2. [file 41598_2025_7398_MOESM2_ESM.docx]

# Supplementary File 2: Oligonucleotides used for quantitative polymerase chain reaction

| **Oligonucleotide-Name** | **Sequence** |
| --- | --- |
| 27 Beta actin forward | 5’-CCTTCCTGGGCATGGAGT-3’ |
| 28 Beta actin reverse | 5’-CAGGGCAGTGATCTCCTTCT-3’ |
| 21 Glucocorticoid receptor alpha forward | 5’-GCAGTGGAAGGACAGCACAA-3‘ |
| 22 Glucocorticoid receptor alpha reverse | 5’-GTCCAACAGTGACACCAGGG-3‘ |
| 19 Glucocorticoid receptor beta forward | 5’-AGCGGTTTTATCAACTGAC-3’ |
| 20 Glucocorticoid receptor beta reverse | 5’-TGAGTTCTATTTTTTGAGCG-3’ |
| 25 Glucocorticoid receptor total forward | 5’-GCAGTGGAAGGACAGCACAA-3’ |
| 26 Glucocorticoid receptor total reverse | 5’-CTCCAACAGTGACACCAGGG-3’ |
