# Supplementary material for: Impact of glucocorticoid receptor polymorphism rs6198 on sepsis survival in a prospective multicenter cohort
Source: Sci Rep. 2025 Jul 9;15:24760. doi: 10.1038/s41598-025-07398-4 (PMC12241491; doi:10.1038/s41598-025-07398-4)
Supplement: Supplementary file 3 — Supplementary Information 3. [file 41598_2025_7398_MOESM3_ESM.docx]

Supplementary File 3: Univariate COX regression analysis that served as the basis for the multivariate model

|  | **Variable** | **Hazard ratio** | **p-value** |
| --- | --- | --- | --- |
| Base characteristics | rs6198 Genotype TT* | 2.45 (1.26-4.75) | **0.008** |
|  | Age | 1.03 (1.00-1.05) | **0.027** |
|  | SOFA Score, day 1 | 1.45 (1.30-1.63) | **<0.001** |
|  | Hydrocortisone therapy** | 2.75 (1.36-5.97) | **0.007** |
|  | SAPS2, day 1 | 1.09 (1.05-1.14) | **<0.001** |
|  | Cardio, day 1 | 1.32 (1.06-1.66) | **0.015** |
| Lab values | Serum lactate (mg/dL) | 1.91 (1.42-2.88) | **<0.001** |
|  | Bilirubin (mg/dL) | 1.45 (0.91-2.39) | 0.123 |
|  | Procalcitonin (PCT) (ng/mL) | 1.01 (1.00-1.04) | 0.118 |
|  | C-reactive protein (CRP) (mg/L) | 1.00 (0.97-1.04) | 0.871 |
|  | Leucocytes (cells/µl) | 0.99 (0.93-1.04) | 0.659 |
