# Supplementary material for: Impact of glucocorticoid receptor polymorphism rs6198 on sepsis survival in a prospective multicenter cohort
Source: Sci Rep. 2025 Jul 9;15:24760. doi: 10.1038/s41598-025-07398-4 (PMC12241491; doi:10.1038/s41598-025-07398-4)
Supplement: Supplementary file 4 — Supplementary Information 4. [file 41598_2025_7398_MOESM4_ESM.docx]

# Supplementary File 4: Interaction Test for rs6198 Genotype and SOFA Score and Hydrocortisone therapy (n=204 patients)

|  | **Variable** | **Hazard ratio** | **p-value** |
| --- | --- | --- | --- |
| Univariate | rs6198 Genotype TT | 1.20 (1.05-1.37) | **0.006** |
|  | SOFA Score ≥9 day 1 | 1.45 (1.30-1.63) | **<0.001** |
|  | Hydrocortisone therapy | 1.22 (1.05-1.40) | **0.007** |
| Interaction | rs6198 Genotype x SOFA Score ≥9 | 1.43 (1.13-1.81) | **0.003** |
|  | rs6198 Genotype x Hydrocortisone therapy | 1.19 (1.03-1.37) | **0.021** |
