# Supplementary material for: Impact of glucocorticoid receptor polymorphism rs6198 on sepsis survival in a prospective multicenter cohort
Source: Sci Rep. 2025 Jul 9;15:24760. doi: 10.1038/s41598-025-07398-4 (PMC12241491; doi:10.1038/s41598-025-07398-4)
Supplement: Supplementary file 5 — Supplementary Information 5. [file 41598_2025_7398_MOESM5_ESM.docx]

# Supplementary File 5: Subgroup description with SOFA Score ≥9 or septic shock at enrolment (n=94), classification according to rs6198 SNP TT and CC/CT-genotype

|  | **TT**  **(n=64)** | **CC/CT**  **(n=30)** | **p-value** |
| --- | --- | --- | --- |
| **Base characteristics** |  |  |  |
| Female sex, n (%) | 21 (33%) | 11 (37%) | 0.816 |
| Age, years (IQR) | 65.0 (57.5-74.5) | 62.5 (54.5-75.5) | 0.652 |
| SAPSII Score, day 1 (IQR) | 36.0 (29.0-44.0) | 34.0 (24.5-43.0) | 0.470 |
| SOFA Score, day 1 (IQR) | 12.0 (10.0-14.0) | 11.0 (10.0-12.0) | 0.104 |
| Ventilatory support*, day 1, n (%) | 57 (89%) | 26 (87%) | 1.000 |
| Length of ICU stay, days (IQR) | 7.26 (2.62-13.69) | 11.89 (4.60-15.07) | 0.126 |
| **Comorbid conditions, n (%)** |  |  |  |
| Hypertension | 39 (61%) | 9 (30%) | 0.121 |
| Cardiovascular disease | 18 (28%) | 6 (20%) | 0.737 |
| Obesity** | 18 (28) | 5 (17%) | 0.882 |
| COPD*** | 5 (8%) | 1 (3%) | 0.990 |
| Diabetes mellitus | 21 (33%) | 5 (17%) | 0.565 |
| Chronic kidney disease | 10 (16%) | 4 (13%) | 0.990 |
| Malignant neoplasms | 14 (22%) | 2 (7%) | 0.322 |
| **Infection focus, n (%)** |  |  |  |
| Pulmonal | 30 (47%) | 11 (37%) | 0.641 |
| Urinary tract | 2 (3%) | 1 (3%) |  |
| Abdomen | 8 (13%) | 5 (17%) |  |
| Central nervous system | 0 (0%) | 1 (3%) |  |
| Bloodstream | 5 (8%) | 1 (3%) |  |
| Skin & Soft Tissue | 6 (9%) | 2 (7%) |  |
| Other/Unknown | 13 (20%) | 9 (30%) |  |
| **Laboratory values, day 1** |  |  |  |
| C-reactive protein [mg/L] | 18.94 (9.66-27.22) | 15.46 (11.49-24.80) | 1.000 |
| Procalcitonin [ng/mL] | 9.93 (1.73-18.25) | 10.32 (3.60-15.66) | 0.844 |
| Leucocytes [1000/µL] | 12.18 (8.76-18.65) | 14.20 (11.75-17.60) | 0.355 |
| Lactate [mmol/L] | 2.00 (1.10-5.26) | 1.45 (1.15-3.40) | 0.349 |
| Creatinine [mg/dL] | 1.32 (0.96-2.22) | 1.41 (1.04-2.03) | 0.879 |
| Bilirubin [mg/dL] | 0.74 (0.37-1.31) | 0.79 (0.46-1.77) | 0.398 |

*Data are presented as n (%) and median (IQR). *Ventilatory support included: CPAP / High flow / Invasive ventilation **defined as BMI≥30kg/m^2^ ***Chronic obstructive pulmonary disease (COPD)*
