# Supplementary material for: Impact of glucocorticoid receptor polymorphism rs6198 on sepsis survival in a prospective multicenter cohort
Source: Sci Rep. 2025 Jul 9;15:24760. doi: 10.1038/s41598-025-07398-4 (PMC12241491; doi:10.1038/s41598-025-07398-4)
Supplement: Supplementary file 6 — Supplementary Information 6. [file 41598_2025_7398_MOESM6_ESM.docx]

# Supplementary File 6: Cox regression analysis for 30d mortality of subgroup with SOFA Score ≥9 or septic shock at enrolment (n=94)

|  | **Variable** | Univariate | | Multivariate | |
| --- | --- | --- | --- | --- | --- |
|  |  | HR | p-value | Hazard ratio | p-value |
| Base characteristics | rs6198 Genotype TT* | 3.60 (1.61-8.07) | **0.002** | 6.16 (1.66-22.80) | **0.007** |
|  | Age | 1.03 (1.01-1.06) | **0.004** | 1.05 (0.99-1.11) | **0.097** |
|  | SOFA Score, day 1 | 1.36 (1.20-1.53) | **<0.001** | 1.12 (0.84-1.48) | 0.438 |
|  | SAPS2, day 1 | 1.08 (1.04-1.12) | **<0.001** | 1.04 (0.96-1.13) | 0.329 |
|  | Hydrocortisone therapy** | 1.47 (0.81-2.65) | **0.007** | 2.43 (0.81-7.32) | 0.113 |
| Lab values | Serum lactate (mg/dL) | 1.24 (1.14-1.34) | **<0.001** | 1.18 (1.00-1.39) | **0.045** |
|  | Bilirubin (mg/dL) | 1.49 (0.92-2.39) | 0.102 | 1.75 (0.92-3.34) | 0.089 |

Multivariate cox regression with Hazard ratios and 95%-CI Intervalls, *Genotype of rs6198-SNP in NR3C1-Gene ** during ICU stay, as described in methods
